# FOXP1 syndrome: a review of the literature and practice parameters for medical assessment and monitoring

**DOI:** 10.1186/s11689-021-09358-1

**Published:** 2021-04-23

**Authors:** Reymundo Lozano, Catherine Gbekie, Paige M. Siper, Shubhika Srivastava, Jeffrey M. Saland, Swathi Sethuram, Lara Tang, Elodie Drapeau, Yitzchak Frank, Joseph D. Buxbaum, Alexander Kolevzon

**Affiliations:** 1 Department of Genetics and Genomic Sciences, Icahn School of Medicine at Mount Sinai, New York, NY USA; 2 Seaver Autism Center for Research and Treatment, Icahn School of Medicine at Mount Sinai, New York, NY USA; 3 Department of Psychiatry, Icahn School of Medicine at Mount Sinai, New York, NY USA; 4 Department of Pediatrics, Icahn School of Medicine at Mount Sinai, New York, NY USA; 5 Children’s Heart Center, Icahn School of Medicine at Mount Sinai, New York, NY USA; 6 Friedman Brain Institute, Icahn School of Medicine at Mount Sinai, New York, NY USA; 7 Mindich Child Health and Development Institute, Icahn School of Medicine at Mount Sinai, New York, NY USA

**Keywords:** FOXP1, Forkhead box protein 1, ASD, Autism spectrum disorder, FOXP1 syndrome

## Abstract

FOXP1 syndrome is a neurodevelopmental disorder caused by mutations or deletions that disrupt the forkhead box protein 1 (*FOXP1*) gene, which encodes a transcription factor important for the early development of many organ systems, including the brain. Numerous clinical studies have elucidated the role of *FOXP1* in neurodevelopment and have characterized a phenotype. FOXP1 syndrome is associated with intellectual disability, language deficits, autism spectrum disorder, hypotonia, and congenital anomalies, including mild dysmorphic features, and brain, cardiac, and urogenital abnormalities. Here, we present a review of human studies summarizing the clinical features of individuals with FOXP1 syndrome and enlist a multidisciplinary group of clinicians (pediatrics, genetics, psychiatry, neurology, cardiology, endocrinology, nephrology, and psychology) to provide recommendations for the assessment of FOXP1 syndrome.

## Introduction

*FOXP1* (forkhead box P1) (OMIM#605515) is a member of the forkhead box family of transcriptional factors that play important roles in the regulation of gene transcription from early development through adulthood [1]. The *FOXP1* gene, located on chromosome 3p14.1, codes for a transcriptional repressor protein. The FOXP1 protein is widely expressed in human tissues and is involved in regulating the development of the brain, heart, lung, esophagus, immune system, and spinal motor neurons [1–7]. It has also been suggested that the FOXP1 protein plays a critical role in striatal regulation and vocal communication, and when disrupted, may contribute to expressive language deficits [8].

In 2009, Pariani and colleagues reported a child with intellectual disability (ID) and a multigenic deletion including the *FOXP1* gene, and hypothesized that *FOXP1* contributed to ID [9]. In 2010, Hamdan and colleagues described two cases with ID and features of autism spectrum disorder (ASD) in whom a deletion involving *FOXP1* and a sequence variant in *FOXP1* were found [10]. Since then, many more cases have been reported and FOXP1 syndrome is now a recognizable entity [11]. FOXP1 syndrome (FOXP1S) is associated with “intellectual disability with language impairment and with or without autistic features” (OMIM #613670) and is caused by *FOXP1* gene deletions and mutations (nonsense, missense, and in-frame deletions) [11].

There have been at least 30 case reports, case series, and cohort studies published in the literature that taken together describe more than 100 individuals with FOXP1S. The majority of information has been collected retrospectively from medical genetic evaluations. The objective of this review is to describe the clinical phenotype of individuals with FOXP1S and establish recommendations to assess the medical, developmental, and behavioral features.

## Methods

We conducted a comprehensive review of the literature published between March 2009, when Pariani and colleagues hypothesized the association of *FOXP1* and ID, and July 2020. We searched for articles on FOXP1S on PubMed and Scopus. Keywords used in PubMed included FOXP1, 3p14.1 [All Fields] and FOXP1 or 3p14.1 and Autism [All Fields], while keywords used in Scopus included FOXP1, 3p14.1 [TITLE-ABS-KEY] and FOXP1 or 3p14.1 and Autism [TITLE-ABS-KEY].

## Results

There were at least ten studies that screened individuals with autism spectrum disorder (ASD) and intellectual disability (ID) for *FOXP1* deletions and mutations. In most of these studies, the clinical features of the patients were not described in detail other than the presence of ASD, learning disability, and/or neurodevelopmental disorder broadly. Other studies screened for *FOXP1* variants in patients with other medical problems such as cardiac and renal malformations [12, 13]. For the purpose of this review, we focused on clinical case reports and case series where insights into the phenotype could be feasibly ascertained.

We identified a total of 62 independent individuals described in 21 case reports and case series, [9–11, 14–31]. Overlapping cases were excluded [32, 33]. Furthermore, one case of a child who had a known *FOXP1* variant and a *PTCH1* pathogenic mutation was also excluded [34]. The data were extracted and sent to multiple clinical specialists for interpretation and to provide multidisciplinary recommendations.

The 62 independent individuals described were predominantly male (male 41: female 21) and ranged in age from 4 months to 31 years old (mean = 11.4 years, SD = 7.1). For most features, 62 cases had neurobehavioral information (Table 1), 59 cases had dysmorphology descriptions (Table 2), and 60 cases had medical histories (Table 3). Unless otherwise specified in the original studies, we used these denominators and assume that if a feature was not recorded, it was absent. We also use these denominators to avoid inflation of prevalence which can affect recommendations for assessment and monitoring.

**Table 1:** Neurobehavioral features associated with FOXP1 syndrome

Clinical features	Pariani et al. [9]	Hamdan et al. [10]	Horn et al. [14]	Carr et al. [15]	Ţuţulan-Cunită et al. [16]	Palumbo et al. [17]	Le Fevre et al. [18]	Lloveras et al. [19]	Dimitrov et al. [20]	Song et al. [21]	Sollis et al., [22]^**a**^	Sollis et al. [23]	Siper et al. [24]	Meerschaut et al. [11]	Myers et al. [25]^**b**^	Johnson et al. [26]^**c**^	Yamamoto et al [27].	Vuillaume et al. [28]	Urreizti et al. [30]	Mutlu-Albayrak et al. [29]	Total (%)
Number of indviduals	1	2	3	1	1	1	1	1	3	1	2	3	9	25	1	1	3	1	1	1	62
Age	1 y 11 m	9 y 11 m, 15 y 11 m	7 y, 5 y 6 m, 6 y	3 y 5 m	5 y	20 y	6 y	2 y 6 m	4 y, 4 m, 1 y	22 y	7 y, 15 y	2 y, 3 y 8 m, 7 y 11 m	5 y - 17 y	5 m - 23 y	4 y 5 m	2 y 11 m	5 y, 8 y, 5 m	31 y	23 y	4 y	mean = 11.4 y; SD = 7.1
Sex	M	1 M, 1F	2 M, 1 F	M	M	M	M	F	1 M, 2 F	F	1 M, 1 F	2 M, 1 F	2 M, 7 F	20 M, 5 F	F	M	2 M, 1 F	F	M	M	41 M:21 F
Deletion (D) or mutation (Mu)	1 D	1 D:1 Mu	3 D	1 D	1 D	1 D	1 D	1 D	3 D	1 Mu	2 Mu	3 Mu	9 Mu	25 Mu	1 Mu	1 Mu	3 D	1 D	1 Mu	1 D	44 Mu:18 D
History of hypotonia	-	-	-	1/1	-	1/1	-	-	-	-	2/2	1/3	8/9	-	1/1	1/1	2/3	1/1	-	-	18/62 (29%)
Motor delays	1/1	2/2	3/3	1/1	1/1	1/1	1/1	1/1	3/3	1/1	2/2	3/3	9/9	22/24	1/1	1/1	3/3	1/1	1/1	1/1	59/61 (97%)
Gait abnormalities	-	-	-	-	-	-	-	-	-	-	-	-	7/9	-	-	1/1	-	-	1/1	-	9/62 (15%)
Speech delays	1/1	2/2	3/3	1/1	1/1	1/1	1/1	1/1	3/3	1/1	2/2	3/3	9/9	23/23	1/1	1/1	2/3	1/1	1/1	1/1	60/60 (100%)
Swallowing or feeding problems	1/1	-	2/3	-	-	-	1/1	-	3/3	-	-	-	3/9	-	1/1	-	2/3	-	-	-	13/62 (21%)
Articulation problems	-	2/2	3/3	1/1	-	-	1/1	-	-	-	2/2	3/3	9/9	9/11	-	-	1/3	-	1/1	-	32/48 (67%)
ID or GDD	1/1	2/2	3/3	1/1	1/1	1/1	1/1	1/1	2/3	1/1	2/2	3/3	8/9	23/24	-	-	2/2	1/1	1/1	1/1	55/61 (90%)
ASD features	-	2/2	-	-	-	1/1	-	-	3/3	-	2/2	-	4/8	14/20	-	-	-	1/1	1/1	-	28/56 (50%)
Behavioral problems	-	2/2	-	-	-	1/1	-	-	-	-	2/2	2/2	9/9	12/19	-	1/1	2/3	-	1/1	-	32/56 (57%)

*ASD* autism spectrum disorder, *D* deletion, *F* female, *GDD* global developmental delay, *ID* intellectual disability, *M* male, *m* months, *Mu* mutation, *SD* standard deviation, *y* years; “-” indicates absent or not reported

^a^Patient 3 was reported by Siper et al. [24]

^b^Patient 1 was reported by Siper et al. [24]

^c^Patient 1 was reported by Myers et al. [25] and Patient 2 did not have enough data

**Table 2:** Dysmorphic features associated with FOXP1 syndrome

Dysmorphic features	Pariani et al. [9]	Horn et al. [14]	Carr et al. [15]	Ţuţulan-Cunită et al. [16]	Palumbo et al. [17]	Le Fevre et al. [18]	Lloveras et al. [19]	Blanco Sánchez et al. [31]	Dimitrov et al. [20]	Song et al. [21]	Sollis et al. [22]	Sollis et al. [23]	Siper et al. [24]	Meerschaut et al. [11]	Myers et al. [25]	Johnson et al. [26]	Yamamoto et al. [27]	Vuillaume et al. [28]	Urreizti et al. [30]	Mutlu-Albayrak et al. [29]	Total (%)
**Head and facial features**																					
Macrocephaly	-	-	-	-	-	1/1	-	-	-	-	-	1/3	4/8	-	-	1/1	-	-	1/1	1/1	9/59 (15%)
Frontal hair upsweep	-	2/3	-	-	-	-	-	-	-	1/1	-	-	2/8	10/24	1/1	-	-	-	-	-	16/59 (27%)
Prominent forehead	-	2/3	1/1	1/1	1/1	1/1	-	1/1	2/3	1/1	1/2	2/3	6/8	23/24	-	1/1	3/3	-	1/1	1/1	48/59 (81%)
Flat midface	-	-	-	-	-	1/1	-	-	1/3	-	-	-	-	-	-	-	-	-	1/1	-	3/59 (5%)
Long philtrum	-	-	-	-	-	-	-	-	-	-	-	-	3/8	-	-	-	-	-	-	-	3/59 (5%)
Short philtrum	-	-	-	1/1	-	-	-	-	1/3	-	-	-	-	-	-	-	2/3	-	-	-	4/59 (7%)
Pointed chin	-	-	-		1/1	-	-	-	-	-	-	-	1/8	9/22	-	1/1	-	-	-	-	12/57 (21%)
**Eyes**																					
Epicanthal folds	1/1	-	-	1/1	-	-	-	-	-	-	-	-	3/8	-	-	-	-	-	-	-	5/59 (8%)
Down slanted palpebral fissures	-	1/3	1/1	-	1/1	1/1	-	1/1	1/3	-	1/2	1/3	-	14/24	-	-	-	-	1/1	1/1	24/59 (41%)
Short palpebral fissures	1/1	-	-	1/1	-	-	-	-	-	-	-	-	-	-	-	-	-	-	-	-	2/59 (3%)
Ptosis	1/1	-	1/1	-	1/1	-	-	-	-	-	-	-	1/8	16/24	-	-	-	-	1/1	1/1	22/59 (37%)
Hypertelorism	-	-	1/1	-	1/1	-	-	-	3/3	-	2/2	1/3	4/8	-	-	-	3/3	-	1/1	1/1	17/59 (29%)
Blepharophimosis	1/1	-	-	-	-	-	-	-	-	-	-	-	-	4/24	-	-	-	-	-	-	5/59 (8%)
Coloboma	-	-	-	-	-	-	-	-	2/3	-	-	-	-	-	-	-	-	-	-	-	2/59 (3%)
**Ears**																					
Malformed ears	-	-	-	1/1	-	-	-	-	2/3	-	-	1/3	-	-	1/1	1/1	2/3	-	1/1	1/1	10/59 (17%)
**Nose**																					
Short nose with broad tip	1/1	1/3	1/1	-	1/1	1/1	-	1/1	2/3	1/1	2/2	2/3	5/8	19/24	-	-	2/3	-	1/1	1/1	41/59 (69%)
Broad nasal bridge	-	-	-	1/1	-	-	-	-	1/3	-	-	-	6/8	-	-	-	-	-	-	-	8/59 (14%)
**Mouth**																					
Thick vermillion	-	-	-	-	1/1	-	-	-	-	-	-	-	3/8	14/24	-	-	-	-	-	-	18/59 (31%)
High-arched palate	-	-	-	-	-	-	-	1/1	1/3	-	-	-	4/8	-	-	-	2/3	1/1	1/1	-	10/59 (17%)
Malocclusion	-	-	-	-	1/1	-	-	-	-	-	-	-	3/8	-	-	-	-	-	-	-	4/59 (7%)
**Extremities**																					
Hyperflexibility of joints	-	-	-	-	-	-	-	-	-	1/1	-	-	1/8	-	-	-	-	-	-	1/1	3/59 (5%)
Clinodactyly	1/1	-	-	-	-	1/1	-	-	-	-	-	-	4/8	6/21	-	-	-	-	1/1	-	13/56 (23%)
Single palmar crease	1/1	-	-	-	-	-	-	-	3/3	-	-	-	2/8	8/21	-	-	-	-	-	-	14/56 (25%)
Prominent finger pads	-	-	1/1	-	-	1/1	-	-	-	-	-	1/3	-	2/22	1/1	-	-	-	-	-	6/57 (11%)
Partial syndactyly 2nd and 3rd toes	-	-	-	-	-	-	1/1	-	-	-	-	-	1/8	-	-	-	-	-	-	1/1	3/59 (5%)
Sandal gap	-	-	-	-	-	-	-	-	2/3	-	-	-	-	-	-	-	-	-	-	-	2/59 (3%)
**Other**																					
Sacral dimple	-	-	-	-	-	-	-	-	2/3	-	1/2	-	1/8	-	-	-	-	-	-	-	4/59 (7%)

“-” indicates absent or not reported

**Table 3:** Medical features associated with FOXP1 syndrome

Medical Features	Pariani et al. [9]	Hamdan et al. [10]	Horn et al. [14]	Carr et al. [15]	Ţuţulan-Cunită et al. [16]	Palumbo et al. [17]	Fevre et al. [18]	Lloveras et al. [19]	Dimitrov et al. [20]	Song et al. [21]	Sollis et al. [22]	Sollis et al. [23]	Siper et al. [24]	Meerschaut et al. [11]	Myers et al. [25]	Johnson et al. [26]	Yamamoto et al [27].	Vuillaume et al. [28]	Urreizti et al. [30]	Mutlu-Albayrak et al. [29]	Total (%)
**Neurology**																					
Spinal defects	-	-	-	-	1/1	-	-	-	-	-	-	-	1/9	2/23	-	-	-	-	-	-	2/60 (3%)
Hypertonia/muscle spasms	1/1	-	-	1/1	-	-	-	-	3/3	-	-	-	-	14/21	-	-	-	-	1/1	-	20/58 (34%)
Abnormal reflexes	-	-	-	1/1	1/1	-	-	-	3/3	-	-	-	-	-	-	-	-	-	-	-	5/60 (8%)
Spastic/contractures	1/1	-	-	-	1/1	-	-	-	3/3	-	-	-	-	10/19	-	-	-	-	1/1	-	16/56 (29%)
Seizures	-	-	-	1/1	-	-	-	-	1/3	-	-	1/3	0/9	3/22	-	-	-	1/1	-	-	7/59 (12%)
Brain abnormalities	1/1 (a)	-	-	1/1 (b)	1/1 (c)	-	1/1 (d)	-	3/3 (e)	-	-	1/2 (f)	6/7 (g)	11/23 (h)	-	1/1 (i)	1/3 (j)	1/1 (k)	-	1/1 (l)	29/58 (50%)
Abnormal EEG	-	-	-	1/1	-	1/1	-	-	-	-	-	-	2/4	-	-	-	-	-	-	-	4/55 (7%)
**Endocrinology**																					
Short stature	1/1	-	-	-	-	-	-	-	3/3	1/2	-	-	-	-	-	-	3/3	-	-	-	8/60 (13%)
Obesity	-	-	2/3	-	-	-	-	-	-	-	-	-	1/9	-	-	-	-	-	-	-	3/60 (5%)
Hypothyroidism	-	-	-	-	-	-	-	-	-	-	-	-	1/9	-	-	-	-	-	-	1/3	2/60 (3%)
Diabetes	-	-	-	-	-	-	-	-	-	-	-	-	1/9	-	-	-	-	-	-	-	1/60 (2%)
**Cardiac**																					
Congenital heart defect	-	-	-	-	1/1 (i)	-	-	1/1 (ii)	3/3 (iii)	-	-	-	2/9 (iv)	9/19 (v)	-	-	1/3 (vi)	-	-	-	17/56 (30%)
**Nephrology/urology**																					
Renal abnormalities	-	-	-	-	-	-	-	-	-	-	-	-	-	1/9 (HK)	-	1/1 (HK)	-	-	1/1 (HK)	-	3/46 (7%)
Micropenis	-	-	-	-	1/1	-	-	-	1/3	-	-	1/3	-	-	-	-	-	-	-	-	3/41 (7%)
Cryptorchidism	1/1	-	-	-	1/1	-	-	-	1/3	-	-	2/3	-	-	-	1/1	1/3	-	1/3	1/3	9/41 (22%)
**Ophthalmology**																					
Visual refractive error	1/1	-	1/3	-	-	-	-	-	-	-	1/2	3/3	5/9	17/21	-	-	-	-	1/1	-	29/58 (50%)
Strabismus	-	-	-	-	1/1	-	-	-	-	-	1/2	3/3	5/9	-	-	-	-	-	-	1/1	11/60 (18%)
Other eye abnormalities	-	-	-	-	-	-	-	-	1/3	-	-	1/3	-	-	-	-	1/3	-	-	-	3/60 (5%)
**Other medical problems**																					
Recurrent infections (ear)	-	-	-	-	-	-	-	-	-	-	-	1/3	6/9	-	-	-	-	-	-	-	7/60 (12%)
Hypoacusis, hearing loss	-	-	-	-	1/1	-	-	-	1/3	-	-	-	1/9	2/11	-	-	3/3	-	-	-	8/48 (17%)
Recurrent upper respiratory tract infections	-	-	-	-	-	-	-	-	2/3	-	-	-	4/9	-	1/1	-	-	-	-	-	7/60 (12%)
Neuroendocrine hyperplasia of infancy	-	-	-	-	-	-	-	-	-	-	-	-	1/9	-	-	-	-	-	-	-	1/60 (2%)
Pulmonary hypertension	-	-	-	-	-	-	-	-	2/3	-	-	-	1/9	-	-	-	1/3	-	-	-	4/60 (7%)
Obstructive sleep apnea	1/1	-	-	-	-	-	-	-	1/3	-	-	-	-	-	-	-	-	-	-	-	2/60 (3%)
Asthma	-	-	-	-	-	-	1/1	-	-	-	-	-	-	-	-	-	-	-	-	-	1/60 (2%)
Gut atresia	-	1/2	-	-	-	-	-	-	-	-	-	-	-	-	-	-	-	-	-	-	1/60 (2%)
Constipation	-	-	-	-	-	-	-	-	-	-	-	-	4/9	-	1/1	-	-	-	-	-	5/60 (8%)
Skin infections	-	-	-	-	-	-	-	-	-	-	-	-	2/9	-	-	-	-	-	-	-	2/60 (3%)
Allergies	-	-	-	-	-	-	-	-	-	-	-	-	2/9	-	-	-	-	-	-	-	2/60 (3%)
Iron deficiency	-	-	-	-	-	-	-	-	-	-	-	-	2/9	-	-	-	-	-	-	-	2/60 (3%)
Sleep issues	-	-	-	-	-	-	-	-	-	-	-	1/3	-	-	-	-	1/3	1/1	-	-	3/60 (5%)
Congenital hip dislocation	-	-	-	-	-	-	-	-	-	-	-	-	-	-	-	-	1/3	-	-	1/1	2/60 (3%)

*a* mild asymmetric enlargement of the ventricles and sulci and minor atrophy; *b* Chiari I malformation (herniation of the cerebellar tonsils through the foramen magnum), hypoplasia of the cerebellar vermis, and dysmorphic corpus collosum; *c* left cerebellum subarachnoid cyst, large cisterna magna, moderate frontal atrophy, and bilateral sclerosis of inner ear bone chain; *d* prominent ventricles; *e* enlarged ventricles, heterotypic nodule and loss of white matter volume, polymicrogyria, fronto-central schizogyria, hypoplastic corpus callosum, and loss of white matter volume; *f* mild widening of extracerebral space; *g* mildly dilated lateral ventricles; non-enhancing subcortical and deep white matter abnormalities; venous angioma in left frontal lobe; prominent Virchow-Robin spaces; small partial cavum septum pellucidum anteriorly; mild diffuse periventricular leukomalacia; arachnoid cysts (cerebellum, left hemisphere); enlarged ventricles; *h* cerebral and/or cerebellar atrophy, cortical, subcortical and deep white matter abnormalities, and/or cerebellar abnormalities; *i* mega cisterna magna; *j* enlarged lateral ventricles; *k* periventricular heterotopia; *l* mega cisterna magna; *i* ASD; *ii* PDA; *iii* ASD, PDA, PS; *iv* PDA, PS, PFO; *v* ASD, PDA, PS; *vi* PDA; *vii* ASD, PDA; *ASD* atrial septal defect; *EEG* electroencephalogram; *HK* horseshoe kidney; *PDA* patent ductus arteriosus; *PFO* patent foramen ovale; *PS* pulmonary stenosis; “-” absent or not reported

### Clinical genetics

In this review, 18 cases had a deletion of the *FOXP1* gene identified by chromosomal microarray analysis (CMA) and 44 had a sequence variant identified by next generation sequencing (Table 1). CMA is used as a first-tier test for individuals with ID, ASD, or developmental delay [35] and identifies individuals with FOXP1S due to 3p14 deletions which include the *FOXP1* gene. The deletions at 3p14 occurred de novo in the vast majority of patients, although in one case, an affected parent with a complex inversion with a breakpoint disrupting *FOXP1* transmitted the rearrangement to an affected child [36]. In another case, a healthy parent carrying a balanced intra-arm (intrachromosomal) insertion transmitted an unbalance rearrangement to an affected child [19].

Fifty-nine cases described in the literature included dysmorphology evaluations. The most common dysmorphic features reported among any cases where exams were performed were prominent forehead (48/59; 81%), short nose with a broad tip or base (41/59; 69%), down slanting palpebral fissures (24/59; 41%), ptosis (22/59; 37%), thick vermillion (18/59; 31%), ocular hypertelorism (17/59; 29%), and frontal hair upsweep (16/59; 27%). Other less common dysmorphic features were single palmar crease (14/56; 25%), clinodactyly (13/56; 23%), pointed chin (12/57; 21%), high-arched palate (10/59; 17%), malformed ears (10/59; 17%), macrocephaly (9/59; 15%), and broad nasal bridge (8/59; 14%) (Table 2).

#### Recommendations

Since CMA does not detect balanced structural chromosome rearrangements, chromosome analysis (karyotype) and/or metaphase fluorescence in situ hybridization (FISH) should be performed in the biological parents to rule out an insertion or inversion which may help to determine recurrence risk. A balanced insertion or inversion involving chromosome 3p14 in a parent significantly increases the risk of recurrence in families, and siblings of the proband should also be tested when relevant. Parental testing is also performed to confirm de novo status. The recurrence risk for future pregnancies is low for apparent de novo aberrations, but it is marginally greater than for the general population (1%) because parents may have germline mosaicism (found specifically within the gamete cells) [35]. Genetic testing for individuals with ID, ASD, or developmental delay, in addition to CMA, involves next-generation sequencing [35], which should be used to test for *FOXP1* sequence variants. Many clinical laboratories offer clinical sequencing (whole exome sequencing and whole genome sequencing) and autism focused sequencing panels, which include the *FOXP1* gene.

**Fig. 1:**
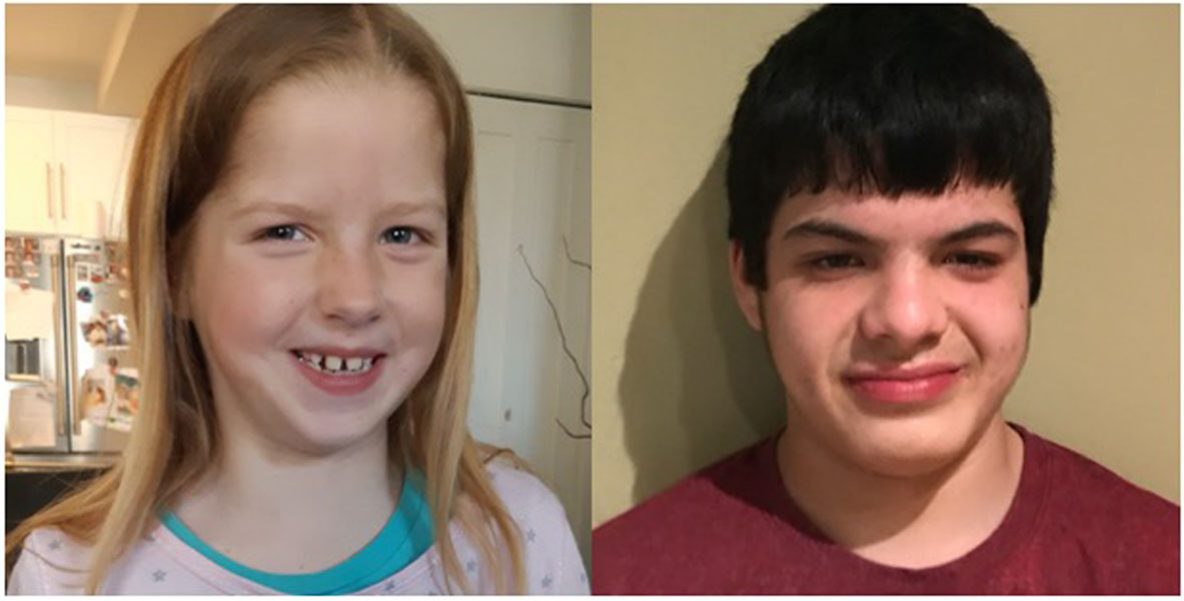
Facial characteristic of two individuals with *FOXP1* sequence variants, ages 9 and 15, including prominent forehead, wide nasal bridge with a broad tip, down slanting palpebral fissures, mild ptosis, thick vermillion, and wide spacing between teeth

Clinical genetics evaluations and dysmorphology exams should be performed to assess growth, head circumference, craniofacial features, pubertal development, and screen for organ malformations (such as congenital heart defects and urogenital abnormalities) and to determine appropriate referrals to other subspecialties. Most patients with FOXP1S have multiple dysmorphic features, although none are specific (Fig. 1).

### Psychology and psychiatry

Mild to moderate ID or global developmental delay was present in 90% (55/61) of cases evaluated and the Full-Scale Intelligence Quotient (FSIQ) ranged from 20 to 93 (mean = 50.04, SD = 9.5). Gross and fine motor delays were present in virtually all cases (59/61; 97%). The mean age of walking unaided for the first time was 24.4 months (range, 16 to 48 months). Speech and language delays were reported in all the cases (60/60; 100%); thus, language impairment may represent a core feature of FOXP1S. Early in life, infants with FOXP1S have hypotonia (18/62; 29%) and feeding issues (13/62; 21%). Early sucking and feeding difficulties may be related to hypotonia. Orofacial hypotonia can also interfere with speech production and cause dysarthria and articulation difficulties later in life. The mean age of first word spoken was 33 months (range, 17 to 42 months). Two thirds of individuals described in the literature had articulation problems (32/48; 67%) (Table 1). Some reports note that expressive language is more impaired than receptive language [12, 37]; however, these findings were largely based on caregiver report rather than standardized assessments. One study prospectively applied norm-referenced assessments (Expressive Vocabulary Test, 2nd Edition; Peabody Picture Vocabulary Tests, 4th Edition for seven and eight children respectively) and the results indicated stronger expressive language skills in comparison to receptive language skills [24].

About 57% (32/56) had documented psychiatric comorbidities, including attention deficit/hyperactivity disorder (ADHD), aggression, obsessive-compulsive traits, mood disorders, and anxiety. ASD symptoms were reported in 50% (28/56) (e.g., social skills deficits, sensory symptoms, and repetitive behaviors/interests). Five of 10 cases assessed using gold-standard diagnostic testing, including the Diagnostic and Statistical Manual for Mental Disorders, Fifth Edition (DSM-5), the Autism Diagnostic Observation Schedule 2 (ADOS-2), and the Autism Diagnostic Interview-Revised (ADI-R) also met criteria for ASD.

#### Recommendations

All patients with FOXP1S should be referred for comprehensive neuropsychological evaluations by licensed clinical psychologists with expertise in the assessment of patients with neurodevelopmental disorders. Evaluations should include measures of cognitive functioning, adaptive behavior, ASD symptomatology, expressive and receptive language, visual-motor integration, academic achievement, executive functioning, and global behavioral functioning (e.g., internalizing and externalizing symptoms). Testing batteries should be tailored to an individual’s age and level of functioning. Evaluations should include a combination of clinician-administered assessments and caregiver report questionnaires. Full re-evaluations are recommended at a minimum of every 3 years to assess progress and update treatment recommendations. Therapists and teachers should measure progress regularly and individuals failing to make expected gains should be evaluated more frequently.

Speech and language therapy, occupational therapy, and behavior therapy are often critical components to treatment plans. We recommend comprehensive evaluations by therapists with experience treating individuals with neurodevelopmental disorders to assess need and frequency of services required. Augmentative and alternative communication evaluations should be provided to all individuals who are nonverbal or minimally verbal. Applied behavior analysis (ABA) is recommended to improve communication, social skills, attention, and learning. ABA should also target activities of daily living and challenging behavior. Families should work with behavior analysts to develop individualized treatment plans. School-age children with FOXP1S often require an individualized education program to ensure an appropriate educational setting, adequate services and appropriate accommodations. In the case of psychiatric comorbidities such as ADHD, anxiety, or irritability/aggression, patients should be referred to a child and adolescent psychiatrist, developmental pediatrician, pediatric neurologist, or geneticist with experience in medication management for individuals with neurodevelopmental disorders.

### Neurology

Hypertonia/muscle spasms (20/58; 34%) and contractures (16/56; 29%) were present in about a third of cases. Contractures of upper extremities can significantly impair activities of daily living and require assessment and treatment spinal defects were also reported (2/60; 3%) (Table 3).

#### Recommendations

A neurology and/or developmental pediatrics referral to evaluate muscle tone, joint mobility, fine and gross motor skills, gait, cranial nerves, and deep tendon reflexes, as well as speech, language, and behavior. These consultations should be followed, as indicated, by appropriate referrals to pediatric physiatry and orthopedics. The possibility of various interventions should be explored including physical and occupational therapy, feeding therapy, orthotics, and bracing. It is important to distinguish between speech delays caused by oromotor hypotonia and language abnormality related to cognitive delays—as both can be present in FOXP1S. Therapies should be initiated as early as possible, and we recommend these interventions begin prior to the onset of missed milestones in cases detected early by genetic methods. Sufficient frequency of interventions is critical to ensure individuals reach their full potential. All children with FOXP1S are candidates for early intervention programs.

### Seizure assessment

Seizures were reported in some cases (7/59; 12%). Details about the seizures were provided in three cases and included staring episodes [15], febrile [23], and tonic-clonic seizures [28]; other reports did not include a description [11, 20]. Among the seven patients with seizures, only four had abnormal findings on electroencephalogram (EEG).

#### Recommendations

If there are clinical concerns about seizures, EEG is recommended. Overnight video-EEG is preferred using the standard 10–20 system with 64 inputs and online automated spike and seizure detection programs. Sedation for the EEG is not generally recommended but can be considered if an EEG cannot be otherwise performed.

Anticonvulsants used for the treatment of seizures in FOXP1S are the same as those used for the treatment of seizure disorders in general. There is insufficient information about the severity of seizures in FOXP1S or about whether certain anticonvulsant medications are more effective than others. EEG can be beneficial in defining the type of seizures and determining treatment. We recommend adopting a low threshold for repeating an EEG with new onset seizures, change in seizure pattern, behavioral changes, or if new neurological signs emerge.

### Brain imaging

Brain abnormalities were evident in about a half of total cases (29/58) although imaging was only explicitly reported in 45 cases. Abnormalities included dilated lateral ventricles, white matter abnormalities, arachnoid cysts, large cisterna magna, corpus callosum defects, moderate frontal atrophy, cerebellar defects, and Chiari I malformation (Table 3).

#### Recommendations

New assessments of individuals affected with FOXP1S should include brain imaging to rule out structural brain defects. Brain imaging may need to be repeated if indicated, such as for new onset or focal neurological symptoms, new seizures, or excessive head growth. Sedation is very frequently needed in children in order to acquire meaningful imaging studies, and the risks of the procedure must be balanced with the potential benefit.

### Endocrinology

Short stature (8/60; 13%) and obesity (3/60; 5%) were described in some patients with FOXP1S. Hypothyroidism (2/60; 3%) and type 2 diabetes (1/60; 2%) were rarely described (Table 3).

#### Recommendations

Endocrine abnormalities should be assessed following the same guidelines for the general population. Emphasis on proper nutrition is essential, especially in the setting of patients with restricted diets or feeding issues. Pubertal development must be clinically evaluated as per the general population screening. Pituitary hormone deficiencies may also be considered because of other brain anomalies such as corpus callosum defects and ventriculomegaly in patients with FOXP1S [38]

### Cardiology

Our review of the literature showed that almost a third of cases (17/56; 30%) had heart abnormalities, including patent ductus arteriosus, patent foramen ovale, pulmonary stenosis, and/or atrial septal defect (Table 3). Chang and colleagues described a patient with atrioventricular septal defect and hypoplastic left ventricle who had a deletion of *FOXP1*; subsequently in 82 patients with atrioventricular septal defect or hypoplastic left heart syndrome, two patients with *FOXP1* mutations were identified (one had hypoplastic left ventricle with mitral valve and aortic valve atresia, and another patient had atrioventricular septal defect, pulmonary atresia and single ventricle in the setting of heterotaxy syndrome) [12].

#### Recommendations

Cardiac defects in patients with FOXP1S may or may not require medical and surgical intervention. A standard cardiac evaluation, including echocardiography and electrocardiography in all patients.

### Nephrology

In our review, genitourinary malformations were reported in some cases, including horseshoe kidney (3/46; 7%), cryptorchidism (9/41 males; 22%), and micropenis (3/41 males; 7%).

This is consistent with findings from Bekheirnia and colleagues which described eight unrelated individuals with de novo mutations in *FOXP1*; four had genitourinary abnormalities, including unilateral renal agenesis, hydronephrosis, hypospadias, and a duplicated renal collecting system [13].

#### Recommendations

Genitourinary tract anomalies may not require universal sonography, but it is indicated for any symptomatic patient (e.g., urinary tract infection; voiding dysfunction) or for those with an externally noted abnormality such as hypospadias.

### Ophthalmology and hearing

Visual refractory issues such as hypermetropia, amblyopia, myopia (29/58; 50%), and strabismus (11/60; 18%) were reported in a significant number of cases. Other severe eye abnormalities (3/60; 5%) included retinitis pigmentosa, cerebral visual impairment, aniridia, microphthalmia, and coloboma. Hearing loss was also reported (8/48; 17%). Recurrent ear infections were reported in some cases (7/60; 12%).

#### Recommendations

Hearing and vision evaluations for all individuals with FOXP1S and corrective measures should be implemented promptly.

### Other medical problems

Other medical problems reported were recurrent upper respiratory infections (7/60; 12%), constipation (5/60; 8%), sleep difficulties (3/60; 5%), hip dysplasia (2/60; 3%), recurrent skin infections (2/60; 3%), and iron deficiency anemia (2/60; 3%). Isolated reports of medical problems included gut atresia (1/60), absent gallbladder (1/60), vocal cord paralysis (1/60), craniosynostosis (1/60), recurrent fever episodes (1/60), asthma (1/60), and neuroendocrine hyperplasia of infancy (1/60) (Table 3).

#### Recommendations

Based on our clinical experience with FOXP1S, some of the described medical problems may be more frequent than noted in the literature, particularly constipation, sleep difficulties, and frequent infections. As such, ongoing monitoring in primary care and appropriate referrals to gastroenterology and sleep specialists, for example, are necessary. Dental problems are also important to assess and are associated with high-arched palate; routine dental care is also recommended.

## Discussion

This literature review revealed a clinically heterogeneous phenotype of FOXP1S. Clinical features consistently reported include global developmental delay/intellectual disability, speech delay and articulation problems, ASD, and mild dysmorphic features. Cardiac, brain, and renal malformations, as well as other medical problems, were reported in some individuals affected by FOXP1S. Severe cardiac defects were uncommon, although studies of individuals with FOXP1S with cardiac malformations are currently underway. Affected individuals are more likely to have hearing deficits, strabismus, visual refractive errors, and recurrent infections. Frequent upper respiratory infections during childhood [24] may be related to the role of FOXP1 in transcriptional regulation of B cell development and T cell suppression [3, 39]. Studies are also currently underway to examine the presence, frequency, and type of infections in children with FOXP1S.

Results from this review are limited by the small number of cases, lack of detailed clinical information, and the variability of assessments used. It is possible that some of the medical features are incidental findings, particularly those observed at a lower rate; further studies are necessary to clarify the phenotype and associated medical problems in FOXP1S.

Robust genotype-phenotype associations have not been reported in FOXP1S. Among cases with the same recurring variant (c.1573C>T, p. Arg525*) different phenotypes have been described. In addition, individuals with *FOXP1* deletions, truncating variants, and missense variants did not have significant differences in the severity of developmental delay [11]. However, individuals with large 3p deletions may be at risk of a more severe clinical presentation as compared to those in whom only the *FOXP1* gene is disrupted [11].

As genetic testing becomes more accessible, the population of individuals with FOXP1S will continue to increase. CMA is a first-tier testing method for individuals with ID and ASD, and sequencing of *FOXP1* is now included in many clinically available neurodevelopmental testing panels. In addition, improved access to whole exome and whole genome sequencing will identify additional cases.

## Conclusions

To date, information about the clinical presentation of individuals with FOXP1S relies mainly on individual case reports and small case series but reveals a heterogeneous phenotype with global developmental delay, intellectual disability, speech impairment, and associated medical features. Advancing knowledge about the clinical features of FOXP1S and providing medical recommendations for assessment is critical for this population. Table 4 includes a summary of the recommended practice parameters for medical assessment. Comprehensive assessment and longitudinal follow up of individuals with FOXP1S are necessary to better understand the clinical phenotype and natural history of FOXP1S and to clarify the efficacy of treatments for affected individuals.

**Table 4:** Recommendations for medical assessments in FOXP1 syndrome

Medical specialty	Clinical features	Assessments
Clinical genetics	Autism spectrum disorder (ASD), intellectual disability (ID), or developmental delay	For affected individuals, chromosomal microarray to identify 3p14 microdeletions; whole exome, whole genome sequencing, or next-generation sequencing panels for ASD and ID For parents, chromosome analysis and fluorescence in situ hybridization to identify rearrangements
	Prominent forehead	Dysmorphology exam and specialty referrals
	Frontal hair upsweep	
	Down slanting palpebral fissures	
	Ptosis	
	Single palmar crease	
	Clinodactyly	
Psychiatry and psychology	Autism spectrum disorder	Gold-standard diagnostic testing
	Intellectual disability	Cognitive and adaptive behavior testing
	Delayed speech, articulation problems	Speech and language evaluation and therapy
	Maladaptive behaviors	Psychiatric evaluation, medication management
Neurology	Seizures	Electroencephalography
	Structural brain abnormalities	Brain imaging and head circumference monitoring
	Feeding difficulties	Feeding therapy evaluation
	Hypotonia	Occupational and physical therapy evaluations
	Motor skill deficits	Physical therapy
Endocrinology	Short stature and obesity	Monitor height, weight, body mass index, and pubertal development
Cardiology	Mild congenital heart defects	Referral to cardiology for echocardiography and electrocardiography
Nephrology	Mild genitourinary malformations	If urinary tract infection or voiding dysfunction, renal ultrasound
Otolaryngology	Recurring ear infections and hearing problems	Hearing test and consider ear tube placement
Ophthalmology	Vision problems	Eye exam
Primary care/pediatrics	Upper respiratory tract infections, gastroesophageal reflux, and constipation	Careful and consistent monitoring and management
Dentistry	High arched palate and malocclusion	Routine dental exams

## Data Availability

This is a literature review. All data generated and analyzed during this study are included in this published article.

## Abbreviations

ADOS-2: Autism Diagnostic Observation Schedule 2
ADI-R: Autism Diagnostic Interview-Revised
ABA: Applied behavior analysis
ASD: Autism spectrum disorder
ADHD: Attention deficit/hyperactivity disorder
BMI: Body mass index
CMA: Chromosomal microarray analysis
DSM-5: Diagnostic and Statistical Manual of Mental Disorders, 5th edition
EEG: Electroencephalography
FOXP1S: FOXP1 syndrome
ID: Intellectual disability
MRI: Magnetic resonance imaging
